# Hepatic proteome network data in zebrafish (*Danio rerio*) liver following dieldrin exposure

**DOI:** 10.1016/j.dib.2019.104351

**Published:** 2019-08-05

**Authors:** Denina B.D. Simmons, Andrew M. Cowie, Jin Koh, James P. Sherry, Christopher J. Martyniuk

**Affiliations:** a University of Ontario Institute of Technology, Faculty of Science, Oshawa, Ontario, L1H 7K4, Canada; b Canadian Rivers Institute and Department of Biology, University of New Brunswick, Saint John, New Brunswick, E2L 4L5, Canada; c Interdisciplinary Center for Biotechnology Research, University of Florida, Gainesville, FL, 32611, USA; d Aquatic Contaminants Research Division, Water Science and Technology, Environment and Climate Change Canada, Burlington, Ontario, L7S 1A1, Canada; e Department of Physiological Sciences and Center for Environmental and Human Toxicology, University of Florida Genetics Institute, College of Veterinary Medicine, University of Florida, Gainesville, FL, 32611, USA

**Keywords:** Pesticide, Proteomics, Biomarker, Aquatics, Hepatotoxicity, Zebrafish, Bioinformatics

## Abstract

Dieldrin is an environmental contaminant that adversely affects aquatic organisms. The data presented in this study are proteomic data collected in liver of zebrafish that were exposed to the pesticide in a dietary exposure. For label free proteomics, data were collected with a quadrupole Time-of-Flight mass spectrometer and for iTRAQ proteomics, data were acquired using a hybrid quadrupole Orbitrap (Q Exactive) MS system. Using formic acid digestion and label free proteomics, 2,061 proteins were identified, and among those, 103 were differentially abundant (p < 0.05 in at least one dose). In addition, iTRAQ proteomics identified 722 proteins in the liver of zebrafish following dieldrin treatment. The label-free approach identified 21 proteins that followed a dose dependent response. Of the differentially abundant proteins identified by iTRAQ, there were 26 unique expression patterns for proteins based on the three doses of dieldrin. Proteins were queried for disease networks to learn more about adverse effects in the liver following dieldrin exposure. Differentially abundant proteins were related to metabolic disease, steatohepatitis and lipid metabolism disorders, drug-induced liver injury, neoplasms, tissue degeneration and liver metastasis. The proteomics data described here is associated with a research article, “Label-free and iTRAQ proteomics analysis in the liver of zebrafish (*Danio rerio*) following a dietary exposure to the organochlorine pesticide dieldrin” (Simmons et al. 2019). This investigation reveals new biomarkers of toxicity and will be of interest to those studying aquatic toxicology and pesticides.

**Table:** Specifications table

Subject area	*Biology*
More specific subject area	*quantitative proteomics, pesticide, hepatotoxicity, biomarkers, fish*
Type of data	*Table, figure, and supplemental file of protein data in excel*
How data was acquired	*Label-free: Mass spectrometry using a quadrupole Time-of-Flight mass spectrometer (Agilent Technologies, Santa Clara, CA, United States of America) iTRAQ: Mass spectrometry using a hybrid quadrupole Orbitrap (Q Exactive) MS system (Thermo Fisher Scientific, Bremen, Germany).*
Data format	Raw and analyzed
Experimental factors	*Samples were digested with trypsin and subjected to SCX fractionation prior to iTRAQ analysis, while for label-free, samples were digested with formic acid with no fractionation prior to reverse phase liquid chromatography separation.*
Experimental features	*Female adult zebrafish,* 5 months *of age were fed dieldrin. There were four treatment groups that included a control, and three doses of dieldrin spiked feed (0.03, 0.15, and 1.*8 μg *DLD/g d.w. feed). Fish were fed for 21 days.*
Data source location	*Laboratory experiment in Saint John, NB, Canada. Proteomics data generated at the University of Florida, Florida, USA and Environment and Climate Change Canada, Burlington, Ontario, Canada*
Data accessibility	*Data are with this article.*
Related Research Article	*D. Simmons, A.M. Cowie, J. Koh, J.P. Sherry, C.J. Martyniuk. Label-free and iTRAQ proteomics analysis in zebrafish liver following dietary exposure to the organochlorine pesticide dieldrin. J. Proteomics. 202, 2019, 103362.*

**Table:** 

**Value of the data** • Identification of biomarker candidates for pesticide-induced hepatotoxicity • Two complementary proteomic methods used to test protein responses following dietary exposure to the legacy pesticide dieldrin, increasing the coverage of the liver proteome. • Discovery of hepatic proteins that are responsive to the organochlorine pesticide dieldrin • Framework for understanding disease networks perturbed by organochlorine pesticides

## 1 Data

Proteomics is used as a tool to identify biomarkers of exposure to environmental contaminants.

Dieldrin is an organochlorine pesticide that can bioaccumulate in fish tissues, resulting in adverse effects within the tissue. These data are proteomics data collected in the liver of zebrafish after being fed the organochloride pesticide dieldrin. These data were collected using two different, but complementary methods (Simmons et al., 2019) [1].

***Label-free relative quantification:*** Label-free data were acquired using a quadrupole Time-of-Flight mass spectrometer. Of 2,061 proteins identified by the database search, 1,563 proteins remained after filtering with the interquantile range estimate (Supplemental Data 1). Among those, 103 proteins (approximately 6.6%) were significantly different in abundance in at least one treatment group compared with the control (p < 0.05). The number of differentially abundant proteins in each of the dieldrin (DLD) treatments [0, LOW = 0.03, MED = 0.15, or HIGH = 1.8 μg/g dieldrin] was: 31 proteins down-regulated and 57 proteins up-regulated in the LOW dose, 27 proteins down-regulated and 11 up-regulated in the MED dose, and 30 proteins down-regulated and 1 proteins up-regulated in the HIGH dose.

***Isobaric tagging for relative and absolute quantitation (ITRAQ)*** data were collected using iTRAQ labelling methodology. Data were acquired using a hybrid quadrupole Orbitrap (Q Exactive) MS system (Thermo Fisher Scientific, Bremen, Germany). There were 772 proteins that were regulated in one or more doses of DLD (Supplemental Data 2). These proteins comprised 26 unique expression patterns for proteins based on the three doses of DLD (Table 1). The remaining proteins that were not changed (3219) are indicated in group X1. The number of differentially expressed proteins that were affected by DLD based on dose was as follows: The LOW DLD treatment resulted in 61 proteins down-regulated and 288 proteins up-regulated, the MED DLD treatment resulted in 99 proteins down-regulated and 185 up-regulated, and the HIGH DLD treatment resulted in 363 proteins down-regulated and 196 proteins up-regulated. The group that contained the highest number of proteins (n = 178) was Expression Pattern XII and these proteins were down-regulated in abundance with the highest dose of DLD. Approximately 18.3% of the proteins detected by iTRAQ were responsive to the dietary exposure (Fold change cut-off of 1.2 fold, p < 0.05).

**Table 1:** Expression patterns in the liver compared to control group (>1.2 or <0.8 with P < 0.05) following dietary exposure to dieldrin.

Expression Type	Low	Medium	High	No. of proteins
1	UP	NO CHANGE	UP	61
2	UP	NO CHANGE	NO CHANGE	51
3	UP	NO CHANGE	DOWN	54
4	UP		UP	34
5	UP		NO CHANGE	14
6	UP		DOWN	6
7	NO CHANGE	UP	UP	14
8	NO CHANGE	UP	NO CHANGE	17
9	NO CHANGE	UP	DOWN	5
10	NO CHANGE	UP	UP	50
11	NO CHANGE	UP	NO CHANGE	3219
12	NO CHANGE	UP	DOWN	178
13	DOWN	NO CHANGE	UP	6
14	DOWN	NO CHANGE	NO CHANGE	25
15	DOWN	NO CHANGE	DOWN	13
16	DOWN	NO CHANGE	UP	1
17	DOWN	NO CHANGE	NO CHANGE	2
18	DOWN	NO CHANGE	DOWN	5
19	NO CHANGE	DOWN	UP	15
20	NO CHANGE	DOWN	NO CHANGE	37
21	NO CHANGE	DOWN	DOWN	57
22	UP	DOWN	DOWN	45
23	UP	DOWN	UP	12
24	UP	DOWN	NO CHANGE	11
25	DOWN	UP	UP	3
26	DOWN	UP	NO CHANGE	6
TOTAL				3941

**Fig. 1:**
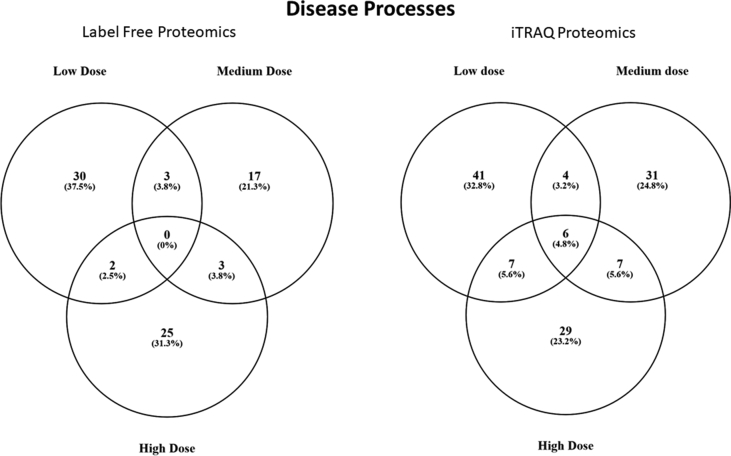
Comparison of disease networks affected in all three doses, using label free proteomics (left graph) and iTRAQ proteomics (right graph).

**Fig. 2:**
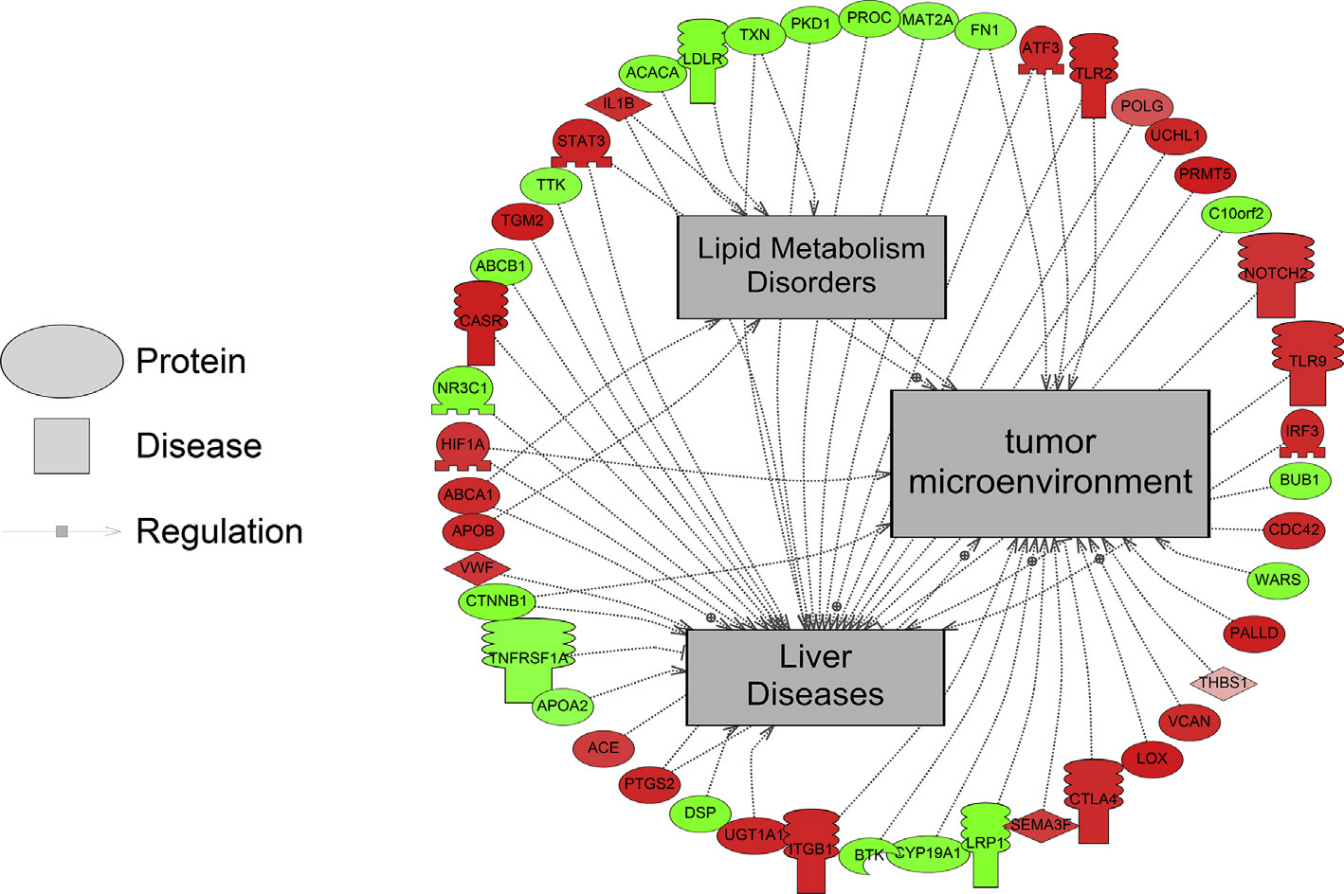
Protein network for lipid metabolism disorders, tumor microenvironment and liver disease following high dose treatment of dieldrin in the zebrafish liver. Protein data were generated using label-free proteomics. Red indicates that protein levels are increased for that protein and green indicates that the protein levels are decreased relative to the control group. The more intense the color, the larger the relative fold change of the protein compared to the control group. Circles indicate proteins, mushroom shaped entities with notches along the side represent receptors, while mushroom-shaped entities with notches on the bottom refer to transcription factors. Abbreviations are provided in Supplemental Data 4.

Examples of diseases associated with proteins quantified in the label free and iTRAQ experiments are presented in Table 2 and all enrichment data are presented in Supplemental Data 3. For label-free proteomics, the number of disease networks identified as differentially expressed based on protein abundance changes in the LOW, MED, and HIGH dose treatments of DLD were as follows: 35, 23, and 30 respectively (Supplemental Data 3). Based on the label-free proteomics, there were no common disease elements identified across all three groups ["LOW DLD", "MED DLD" and "HIGH DLD"] but there were some overlapping diseases in two of the three doses (Fig. 1). In the LOW DLD group, proteins related to lymphoma (T-cell), growth retardation, severe combined immunodeficiency, metabolic diseases, neoplasms and pathologic processes were decreased in protein abundance in the liver. In the MED DLD group, edema and calcification were disease networks that were decreased in abundance in fish liver while upregulated networks included wounds, multiple organ failure, hyperglycemia, liver neoplasms, and iron overload. In the HIGH DLD group, lipid metabolism disorders, neoplastic processes, and proteins involved in healing impairment were down-regulated as a network, while networks associated with growth retardation, tumor microenvironment, and liver diseases were increased in the liver (Table 2, Supplemental Data 3). One theme that emerged from all three treatment groups was the dysregulation of proteins related to tumor and liver disease (Fig. 2).

**Table 2:** Examples of enriched disease sub-networks related to protein profiles in the liver. The complete list is provided for each dose in Supplemental Data 2 and 3. Disease are organized by median fold change within each treatment. LOW = 0.03, MED = 0.15, and HIGH = 1.8 μg/g DLD in pelleted feed.

Method	Dose	Gene Set Seed	# of Measured Proteins	Median change	p-value
Label-Free	Low	Precursor Cell Lymphoblastic Leukemia-Lymphoma	16	−9.48	0.011
		acute hepatitis	7	−9.48	0.029
		Hematologic Diseases	11	−9.10	0.036
		Metabolic Diseases	32	−7.24	0.047
		Neoplasms	218	−5.45	0.048
		Pathologic Processes	54	−5.37	0.048
		leukemogenesis	18	−4.46	0.016
		arterial thrombosis	8	−3.96	0.034
Label-Free	Medium	calcification	17	−5.16	0.036
		Edema	25	−4.07	0.016
		Hemorrhage	27	1.15	0.037
		Hyperglycemia	23	2.21	0.022
		Liver Neoplasms	11	3.34	0.050
		Iron Overload	8	3.87	0.049
		Multiple Organ Failure	5	6.33	0.018
		cartilage degeneration	10	6.33	0.042
		Venous Thrombosis	6	7.95	0.018
Label-Free	High	Lipid Metabolism Disorders	5	−11.02	0.016
		healing impairment	5	−5.83	0.025
		Neoplastic Processes	5	−5.06	0.022
		Liver Diseases	31	3.40	0.039
		Lymphoma, B-Cell	8	3.47	0.023
		tumor microenvironment	21	4.43	0.042
		Leukemia, Myeloid	12	5.59	0.028
iTRAQ	Low	Neoplasm, Residual	7	1.84	0.013
		cartilage loss	18	1.84	0.018
		Neuroendocrine Tumors	9	2.08	0.009
		Influenza, Human	20	2.15	0.028
		Adenoviridae Infections	10	2.15	0.028
		liver metastasis	27	2.80	0.017
		Neoplasm Micrometastasis	9	2.80	0.048
		brain metastasis	14	2.86	0.001
		Sarcoma, Synovial	5	2.91	0.048
		Congenital Disorders of Glycosylation	6	3.13	0.019
		intermittent hypoxia	6	5.80	0.012
		Carcinoma, Ductal	8	5.80	0.023
iTRAQ	Medium	recurrent infection	6	−2.94	0.017
		Weight Loss	54	−1.61	0.001
		Drug-Induced Liver Injury	6	−1.39	0.044
		Steatohepatitis	86	−1.34	0.031
		Liver Failure, Acute	17	−1.15	0.008
		Retroviridae Infections	11	−1.09	0.001
		neuropsychiatric manifestations	8	−1.07	0.036
		Acquired Immunodeficiency Syndrome	7	1.59	0.038
iTRAQ	High	Neoplasm, Residual	7	−15.85	0.003
		Coloboma	5	−15.85	0.030
		Connective Tissue Diseases	5	−8.47	0.039
		smooth muscle hypertrophy	6	−7.38	0.003
		tissue degeneration	6	−7.38	0.023
		recurrent infection	6	−6.79	0.025
		Protein Deficiency	10	−3.24	0.042
		Hyperhomocysteinemia	13	−3.10	0.049
		epithelial damage	16	−2.78	0.004
		liver metastasis	27	−2.38	0.013
		teratogenesis	12	−2.25	0.019
		tumor spheroid	27	−1.64	0.020

**Fig. 3:**
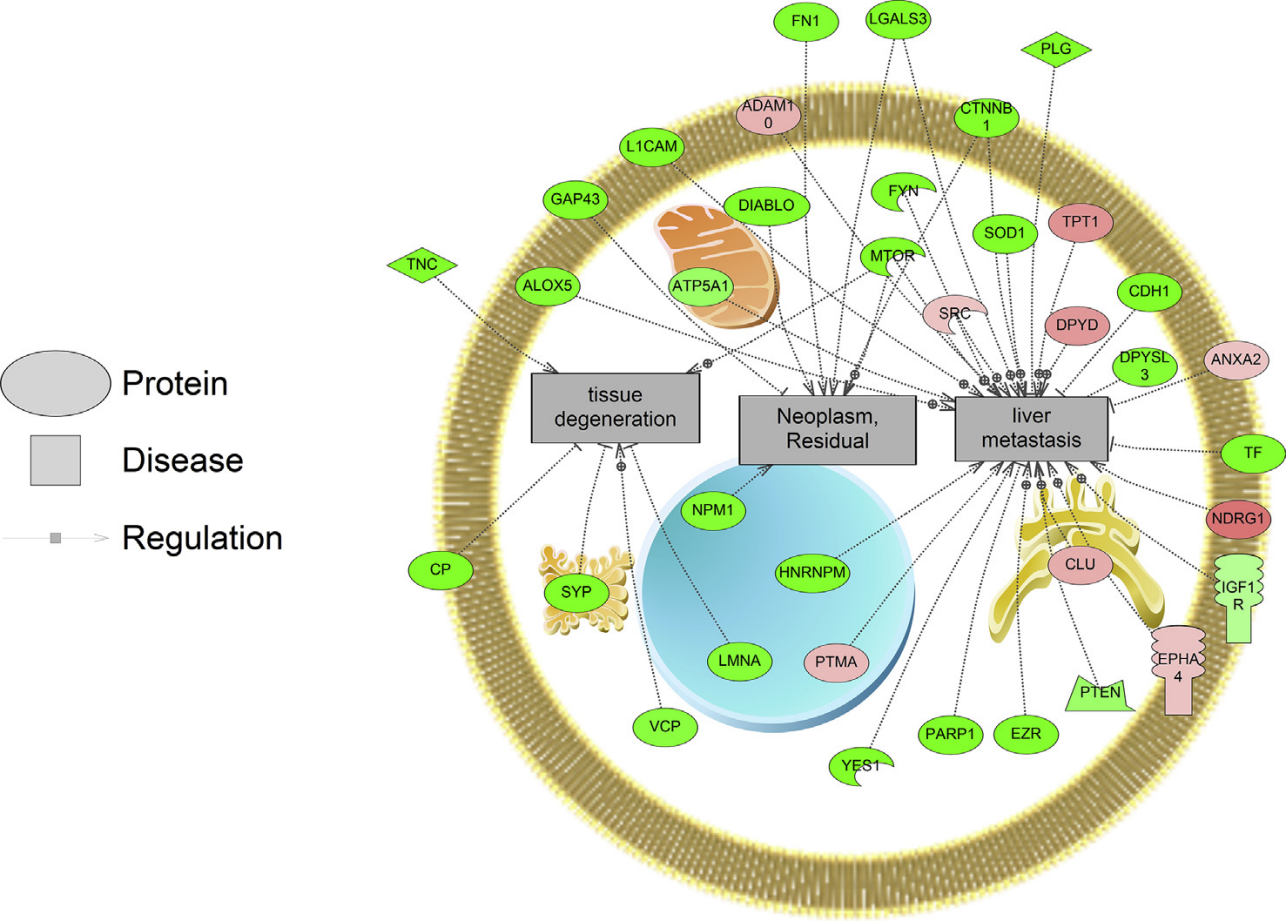
Protein network for tissue regeneration, neoplasms, and liver metastasis. Protein data were generated using iTRAQ proteomics. Red indicates that protein levels are increased for that protein and green indicates that the protein levels are decreased relative to control. The more intense the color, the larger the relative fold change of the protein compared to the control group. Circles indicate proteins, mushroom shaped entities with notches along the side represent receptors, while mushroom-shaped entities with notches on the bottom refer to transcription factors. Abbreviations are provided in Supplemental Data 4.

**Fig. 4:**
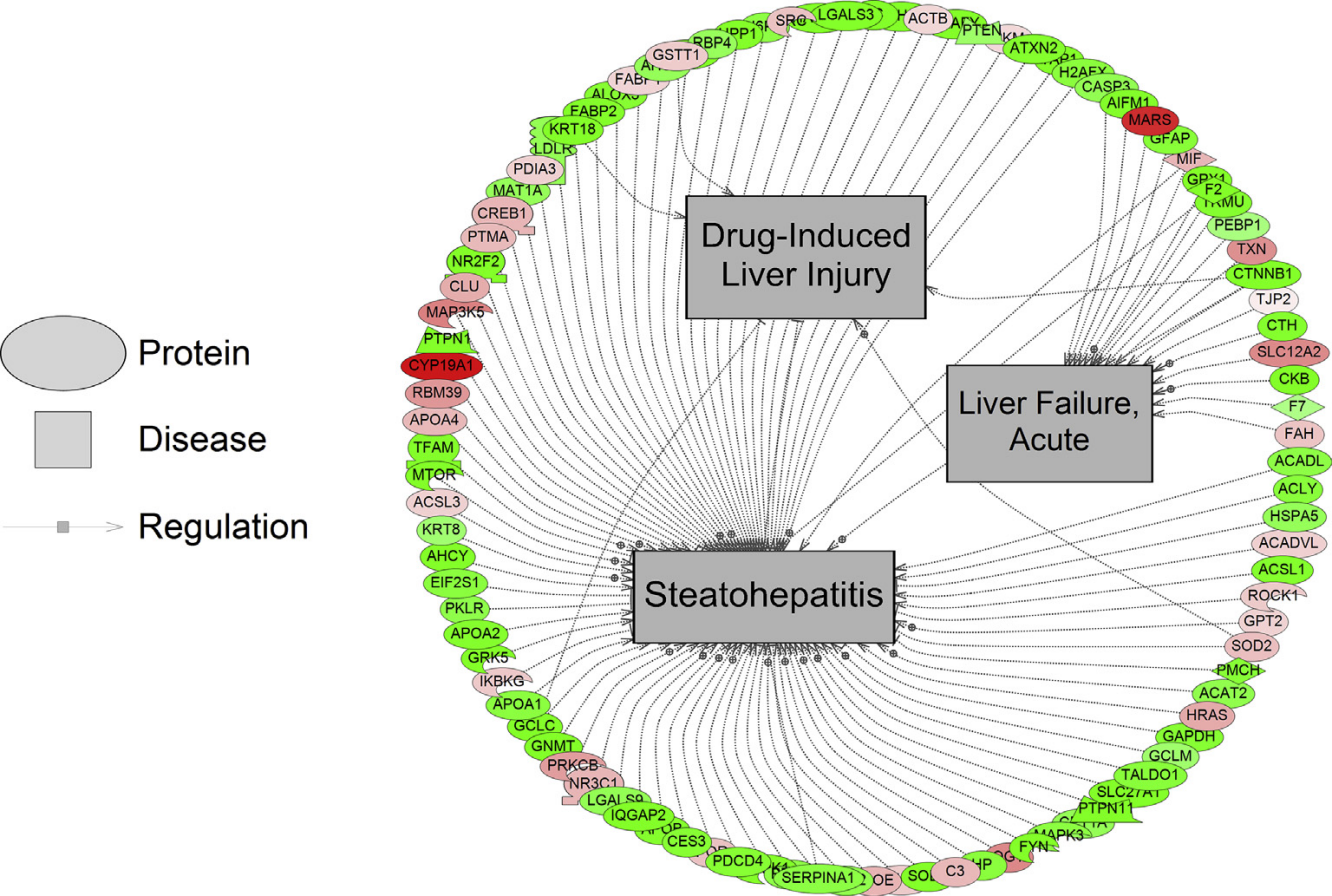
Protein network for drug induced liver injury, acute liver failure and steatohepatitis. Protein data were generated using iTRAQ proteomics. Red indicates that protein levels are increased for that protein and green indicates that the protein levels are decreased relative to control. The more intense the color, the larger the relative fold change of the protein compared to the control group. Circles indicate proteins, mushroom shaped entities with notches along the side represent receptors, while mushroom-shaped entities with notches on the bottom refer to transcription factors. Abbreviations are provided in Supplemental Data 4.

For iTRAQ proteomics, the number of disease networks identified as differentially expressed based on protein abundance changes in the LOW, MED, and HIGH dose treatments of DLD were as follows: 58, 48, and 49 respectively (Supplemental Data 3). Based on the iTRAQ proteomics, there were 6 common disease elements identified across all three groups ["LOW DLD", "MED DLD" and "HIGH DLD"] (Fig. 1). These six common disease networks, affected independent of dose were seizure susceptibility, reflex epilepsy, mucosal damage, cerebellar ataxia, cartilage loss, and erythema. In the LOW DLD group, proteins related to Ehlers-Danlos Syndrome were decreased while proteins related to neoplasm micrometastasis, carcinoma (ductal), and residual neoplasm were increased in protein abundance in the liver. In the MED DLD group, weight loss, acute liver failure, recurrent infection, and drug induced liver injury were disease networks that were decreased in abundance in fish liver while upregulated networks included acquired immunodeficiency syndrome and anxiety disorders. In the HIGH DLD group, residual neoplasms, epithelial damage, liver metastasis, and tissue degeneration were down-regulated as a network, while a network associated with invasive breast care was increased in the liver (Supplemental Data 3). The large majority of the protein networks in the liver were suppressed with DLD exposure with the highest dose. Example networks for disease processes are shown in Fig. 3, Fig. 4. Lastly, five disease networks (in one or more doses) were in common between the label free and iTRAQ proteomics experiments; endometrial carcinoma, bronchopulmonary dysplasia, stomach ulcer, cartilage degeneration, and brain metastasis.

## 2 Experimental design, materials and methods

### 2.1 Protein extraction, digestion, and iTRAQ labeling

For label-free, peptides were prepared from liver protein extracts for each individual liver extract (n = 7). Proteins were re-suspended in 100 mM TEAB (final concentration 20 mg protein/ml). The proteins were then reduced and acetylated using 100 mM tris(2-carboxyethyl)phosphine and 200 mM 2-iodoacetamide. Proteins were digested with 10% v/v formic acid for 30 min at 115 °C. Peptide digests were then evaporated to near dryness and re-suspended in 0.1% formic acid and 5% acetonitrile.

For iTRAQ, proteins were dissolved in denaturant buffer (0.1% SDS (w/v)) and dissolution buffer (0.5 M triethylammonium bicarbonate, pH 8.5) in the iTRAQ Reagents 8-plex kit (AB sciex Inc., Foster City, CA, USA). For each sample, 60 μg of each protein was reduced, alkylated, trypsin-digested, and labeled according to the manufacturer's instructions (AB Sciex Inc.). The liver were labeled as follows: (control was labelled with 117 and the three treatments were labelled as LOW = 118, MED = 119, and HIGH dose = 121). This was done for three independent biological replicates/group. Thus, there were 3 iTRAQ experiments conducted.

### 2.2 LC-MS/MS analysis

For label-free proteome analysis, the peptides were analyzed by liquid chromatography–tandem mass spectrometry (LC–MS/MS). Peptides were separated on an Agilent 1260 Infinity nano-HPLC-Chip cube system, using a ProtID chip 150 II (300 Å C18 150 mm) with a thermostat-controlled column temperature of 40 °C and an autosampler chilling temperature of 8 °C. Both the capillary and the nano-pump timetables were: 0–45 min 0–60% solvent B, 45–50 min 90% solvent B, 50–60 min 0% solvent B. The inner valve was switched to the capillary pump at 45 min. The Agilent 6520 Accurate-Mass Quadrupole Time-of-Flight (Q-TOF) was used as the detector in tandem to the Agilent 1260 system. Samples were ionized using by z-spray with: positive polarity, gas temperature 325 °C, gas flow 5 L/min, capillary 1950 V, fragmentor 180 V, skimmer 65 V, and octopole RF of 750. Scan was performed in Auto MS/MS mode with an MS range of 300–3200 Da and scan rate of 1 cycle/s and an MS/MS range of 50–3200 Da and scan rate of 1 cycle/s using a collision energy ramp with slope 3.7 and offset 2.5. Ten precursors per cycle were the maximum. The absolute threshold to trigger MS/MS scan was an absolute intensity of 1000 counts or a relative threshold of greater than 0.05% total intensity counts. Active exclusion was enabled after 2 spectra were collected for 6 seconds. Reference ion masses of 322.048121, 1221.990637, and 2421.91399 were used for simultaneous mass axis calibration throughout the analysis. Each analytical run included a blank, a peptide standard, and a BSA digest standard, which were injected every 10 samples for quality assurance. Samples were injected once per individual.

For iTRAQ proteomes, labeled peptides were desalted with C18-solid phase extraction and dissolved in strong cation exchange (SCX) solvent A (25% (v/v) acetonitrile, 10 mM ammonium formate, and 0.1% (v/v) formic acid, pH 2.8). The peptides were fractionated using an Agilent HPLC 1260 with a polysulfoethyl A column (2.1 × 100 mm, 5 μm, 300 Å; PolyLC, Columbia, MD, USA). Peptides were eluted with a linear gradient of 0–20% solvent B (25% (v/v) acetonitrile and 500 mM ammonium formate, pH 6.8) over 50 min., followed by ramping up to 100% solvent B in 5 min. The absorbance at 280 nm was monitored and a total of 14 fractions were collected. The fractions were lyophilized and resuspended in LC solvent A (0.1% formic acid in 97% water (v/v), 3% acetonitrile (v/v)). A hybrid quadrupole Orbitrap (Q Exactive) MS system (Thermo Fisher Scientific, Bremen, Germany) was used with high energy collision dissociation (HCD) in each MS and MS/MS cycle. The MS system was interfaced with an automated Easy-nLC 1000 system (Thermo Fisher Scientific, Bremen, Germany). Each sample fraction was loaded onto an Acclaim Pepmap 100 pre-column (20 mm × 75 μm; 3 μm-C18) and separated on a PepMap RSLC analytical column (250 mm × 75 μm; 2 μm-C18) at a flow rate at 350 nl/min during a linear gradient from solvent A (0.1% formic acid (v/v)) to 25% solvent B (0.1% formic acid (v/v) and 99.9% acetonitrile (v/v)) for 80 min, and to 100% solvent B for additional 15 min.

### 2.3 Spectral processing and protein identification

Label-free proteins were identified by search against both the Uniprot and NCBI Teleostei subset protein database (downloaded on Feb 13, 2014; 729,330 entries). Spectral files were grouped into folders by treatment and each folder was searched separately using Spectrum Mill Software (Version A.03.03 SR4). Peptides were validated manually and accepted when at least one peptide had a peptide score (quality of the raw match between the observed spectrum and the theoretical spectrum) greater than 5 and a %SPI (percent of the observed spectral intensities that are accounted for by theoretical fragment peaks) of greater than 60% (recommended criteria for data obtained by an Agilent Q-TOF mass spectrometer). Data were statistically analyzed using the open source online software MetaboAnalyst 4.0 [2]. Data were treated as follows: missing values were replaced with a small number using the default setting, data were filtered using the interquantile range estimate, and then transformed using median normalization and Pareto scaling. Fold change was determined for each treatment by performing a volcano plot. ANOVA with Fisher's LSD was conducted to determine whether treatments differed in protein abundance. Adjusted p-values (q-values) were calculated but not used for filtering. Pattern searching correlation analysis was used to determine which molecules demonstrated a significant positive or negative dose-response relationship.

For iTRAQ, the raw MS/MS data files were processed by a thorough database searching approach considering biological modification and amino acid substitution against the National Center for Biotechnology Information (NCBI) Teleostei database (downloaded on Feb 13, 2014; 729,330 entries) using the ProteinPilot v4.5 with the Fraglet and Taglet searches under ParagonTM algorithm [3]. The following parameters were considered for all the searching: fixed modification of methylmethane thiosulfonate-labeled cysteine, fixed iTRAQ modification of amine groups in the N-terminus, lysine, and variable iTRAQ modifications of tyrosine. For protein quantification, only MS/MS spectra that were unique to a particular protein and where the sum of the signal-to-noise ratios for all the peak pairs >9 were used for quantification. The accuracy of each protein ratio is given by a calculated error factor from the ProGroup analysis in the software, and a P value is given to assess whether the protein is significantly differentially expressed. The error factor is calculated with 95% confidence error, where it is the weighted standard deviation of the weighted average of log ratios multiplied by Student's t factor. The P value is determined by calculating Student's t factor by dividing the (weighted average of log ratios – log bias) by the weighted standard deviation, allowing the determination of the P value with n) 1° of freedom, where n is the number of peptides contributing to the protein relative quantification (software default settings, AB Sciex, Inc.). To be identified as being significantly differentially expressed, a protein had to contain at least three spectra (allowing the generation of a P value), with P < 0.05. Additivity of protein expression was assessed quantitatively; we calculated additive expression based on mid-parent values (MPVs; averaged values from two biological replicates of each parent). To be identified as being significantly differentially expressed, a protein was quantified with at least three unique spectra in at least two of the biological replicates, along with a Fisher's combined probability of <0.05 and a fold change of ±1.2.

The MS proteomics data have been deposited in the ProteomeXchange Consortium [4] via the MassIVE partner repository with the data set identifier PXD014622 and MSV000084091 and PXD014480.

### 2.4 Protein network analysis

For proteomics network analysis with Pathway Studio (v11) (Elsevier), all proteins quantified in the liver with label-free and iTRAQ proteomics were imported into the program separately using Name + Alias (i.e. mammalian homologs were identified for the zebrafish proteins). Each dose was analyzed separately for disease networks and subnetwork enrichment analysis was based on 1000 permutations of fold change data using a Kolmogorov–Smirnov test. The default option of “best p-value, highest magnitude of response” was used to map protein networks. The enrichment p-value for all queries was set at p < 0.05. Subnetwork enrichment analysis was conducted in Pathway Studio for the list of proteins, and diseases were queried. These disease networks represent those that are significantly represented by proteins that are differentially regulated by dieldrin. All abbreviations for the network are provided in Supplemental Data 4.
